# Thyroid abnormalities in acromegaly in the era of improved disease control: a two-stage study

**DOI:** 10.1007/s40618-026-02919-y

**Published:** 2026-05-22

**Authors:** Luciana P. S. Diniz Leite, Beatriz S. Soares da Rocha, Gustavo A. P. Diniz Leite, Gabriel A. P. Diniz Leite, Juliana Beaudette Drumond, Lucas G. de O Freitas, Izabella S. Freitas, Ângela C. Leal, Nathalie O. Santana, Adriana M. Lamego Rezende, Enaldo V. Melo, Arthur M. Pereira Oliveira, Elenilde G. Santps, Viviane C. Campos, Roberto Salvatori, Manuel H. Aguiar-Oliveira

**Affiliations:** 1 Division of Endocrinology, Health Sciences Graduate Program, Federal University of Sergipe, Aracaju, 49060–100 Sergipe Brazil; 2 Department of Endocrinology of the Federal University of Minas Gerais, Belo Horizonte, 30130-100 MG Brazil; 3 Department of Medical Imaging of the Federal University of Minas Gerais, Belo Horizonte, MG Brazil; 4 Faculty of Medicine, Federal University of Minas Gerais, Belo Horizonte, 30130-100 MG Brazil; 5 Statistics division, Health Sciences Graduate Program, Federal University of Sergipe, Aracaju, 49060–100 Sergipe Brazil; 6 Division of Neurosurgery, Hospital de Cirurgia, Aracaju, Sergipe, 49055-000 Brazil; 7 Health Sciences Graduate Program, Federal University of Sergipe, Aracaju, 49060– 100 Sergipe Brazil; 8 Division of Endocrinology, Diabetes and Metabolism, Department of Medicine, The Johns Hopkins University School of Medicine Baltimore, Baltimore, MD 21287 USA; 9 Division of Endocrinology, Federal University of Sergipe, Street Claudio Batista s/n, Aracaju, 49060–100 SE Brazil

**Keywords:** Acromegaly, Thyroid gland, Growth hormone, Insulin-like growth factor

## Abstract

**Purpose:**

Acromegaly is associated with increased risk of diffuse or nodular goiter and possibly of thyroid cancer. The aim of this study was to verify whether these findings persist in the current scenario of improved disease control.

**Methods:**

A two-steps protocol was carried out:. In the first (retrospective), clinical and laboratory data (including 969 IGF-I measurements), and the frequency of thyroid cancer were recorded for 92 acromegaly patients over a 28-year period. The annual control percentage was defined as the average of the ratios between each of the 969 IGF-I measurements over the total follow-up period and the respective upper age limit (IGF-I/ULN) less than 1.Acromegaly activity was determined at the last visit. In the second step (cross-sectional), 74 of the 92 acromegaly patients underwent thyroid ultrasound and were subjected to a two-stage cluster analysis, with the aim of grouping them into homogeneous clusters.

**Results:**

70% of the 92 patients had inactive disease, with a median control rate of 50%, with four cases of thyroid cancer (4.3%), only one in a patient with active disease. Two-thirds of the 74 patients (evaluated by ultrasound) had nodules, and 27% had diffuse thyroid hyperplasia. Three clusters were segregated with high significant differences in sex and echotextural alterations (*p* < 0.0001 in both cases). Age, number of nodules, thyroid volume and follow-up time were similar between the clusters.

**Conclusion:**

No inherent thyroid changes were apparently identified in a large cohort of acromegaly patients. Better control during follow-up may attenuate thyroid manifestations in acromegaly.

## Introduction

There are several interactions between the somatotropic axis and the TSH-thyroid axis. Thyroid hormones have a fundamental role in the initiation and maintenance of somatic growth and play an important role in regulating GH secretion and action [1, 2, 3, 4]. On the other hand, both systemic and local insulin-like growth factor (IGF)-I and IGF-II are important proliferative factors for thyrocytes, increasing the stimulatory effect of TSH [5–9]. Accordingly, individuals with severe untreated GH deficiency due to a homozygous mutation in the GHRHR gene have smaller thyroid volume, confirming that GH plays a permissive role in thyroid gland growth [10]. Conversely, acromegaly is characterized by thyroid follicular cells hyperstimulation due to proliferative effects of GH and IGF-I [11, 12, 13, 14, 15, 16, 17]. Not surprisingly, diffuse thyroid hyperplasia, thyroid nodules and hyperthyroidism, are common in acromegalic patients [13, 18, 19], but with divergent data for thyroid cancer [11, 14, 20, 21], with some studies showing a higher frequency [16, 22–26], not shown in other [27–29]. Two Brazilian studies published more than a decade ago [30, 31] suggested a higher prevalence of diffuse thyroid hyperplasia, nodules and thyroid cancer in acromegaly compared with the general population. However, since then the medical care of patients with acromegaly improved substantially, with all patients having access to drug treatments through the unified health system (*Sistema Unico de Saúde*). The aim of this study was to verify whether this higher frequency of thyroid alterations persists in the current scenario of acromegaly treatment in Brazil, with greater availability of treatment and a possibly better control rate.

## Subjects and methods

### Design

A two-step protocol was performed: the first one epidemiological and the second one ultrasonographic. The current study was approved by the Federal University of Sergipe Institutional Review Board (CAAE UFS 83898424.9.1001.5546.,Opinion Number° 7.214.885).

#### Experiment 1

In this retrospective study, medical records of 105 acromegalic patients were analyzed over 28 years from two tertiary referral centers in Brazil, 22 patients from the neuro-endocrinology division of the University Hospital of Federal, University of Sergipe (HU–UFS), in Aracaju, and 83 from the neuro-endocrinology division the University Hospital (*Hospital das Clínicas*) of the Federal University of Minas Gerais (HC-UFMG), in Belo Horizonte. Thirteen patients were excluded due to incomplete data (*n* = 8), age at diagnosis less than 18 years (*n* = 3), multiple endocrine neoplasia (*n* = 1), and prior thyroid lobectomy (*n* = 1), leaving 92 subjects. Clinical and laboratory data were extracted from the institutional electronic medical registry, using standardized data collection spreadsheets. Date of birth, sex, height, weight, body mass index (BMI), type of treatment, medication use, surgery history, current and previous pathologies, specific clinical condition, blood glucose, serum GH, IGF-I, free T4 (fT4) and TSH were registered. The annual control percentage was defined as the average of the ratios between each of the 969 IGF-I measurements over the total follow-up period and the respective upper age limit (IGF-I/ULN) less than 1.Acromegaly activity was determined at the last visit. Disease activity was defined when the ratio between each patient’s last IGF-I measurement and their respective upper limit of normal (IGF-I/ULN) was greater than 1. Each patient’s current treatment was also recorded. In this way, we have an evaluation of each patient’s film throughout the entire follow-up, culminating in the impactful final scene. Data of this experiment are descriptive and were compared with international and Brazilian studies.

#### Experiment 2

In this transversal experiment, the 92 acromegaly patients were recruited via phone call or WhatsApp messages. Due to logistical reasons (mainly the distance from homes), we were able to exam seventy-four people. An ultrasonographic exam was performed by the same trained operator (LP de SD L) in both centers, with the patient in the supine position with neck hyperextension At UFMG, the examinations were performed in Belo Horizonte, at the Radiology Center of the Hospital das Clínicas (West Wing, Room 4), using a Toshiba Precision Aplio MX system (Otawara, Japan) with a multifrequency linear transducer (L6–12 MHz). At UFS, the examinations took place at the Diagnostic Support Service (SADT) of the University Hospital in Aracaju, using Philco Affiniti 70 equipment (Eindhoven, Netherlands) with a multifrequency linear transducer (L5-18 MHz). Thyroid volume was defined by measuring the longitudinal, anteroposterior, and transverse diameters, applied to the ellipsoid volume formula for each lobe and the isthmus (lobe volume (cm³) = longitudinal x anteroposterior x transverse x π/6 (correction factor). Total thyroid volume was calculated as the sum of the volumes of the thyroid lobes and the isthmus. A low-frequency convex transducer was used when it was not possible to measure the lobe in a single acoustic window. The thyroid volume considered normal was 12–18 mL for men and 10–15 mL for women. Echotextural patterns were characterized as homogeneous or heterogeneous, and parenchymal echogenicity was described as preserved or reduced compared to the submandibular glands. Thyroid nodules were classified in presence, location, number, and malignancy risk classification using the ACR-TIRADS score [32].

### Statistical analysis

Kolmogorov–Smirnov and Shapiro–Wilk tests were used to test the normality of the variables. Variables with normal distribution were expressed as mean (standard deviation), variables with non-normal distribution were expressed as median (interquartile range), and categorical variables were expressed as n (percentage). Pearson’s chi-square test was used to check for frequency differences in nodule size according to disease activity. In this article, we also use cluster analysis, which is the art of finding groups in data [33]. Cluster analysis is a multivariate analysis that allows the identification of groups with homogeneous characteristics and can be used when there are at least three numerical variables. This technique disaggregates a set of objects into smaller subsets (clusters) according to their characteristics (variables). Through mathematical distance calculations, it is possible to assign a measure of proximity (similarity) between each object and the cluster. Thus, clusters are formed so that the distances between the members of a cluster are minimal and the distances between the clusters are maximal [33]. The two-step cluster analysis method was used with categorical (sex and ecotextural changes) and continuous variables (age, number of thyroid nodules, thyroid volume, follow-up time and percentage of control). The number of clusters to be formed was not specified beforehand. Statistical analysis was performed using IBM SPSS (Statistical Package for the Social Sciences), version 23.0, Chicago, IL, USA). The accepted level of significance was less than 0.05, and all tests were two-tailed.

## Results

Table 1 shows clinical data of 92 patients, with a range of ages between 23 and 90 years,71.7% women and 81.5% with macroadenomas. Three-quarters of them were prediabetic or diabetic, and three-fifths had hypertension. Papillary thyroid cancer occurred in only two first-degree relatives of the patients. Most patients had undergone surgery, either alone (31.5%) or in combination with medications (57.6%). The annual control rate was 50 (50), median (interquartile range), with a range from 0 to 100%.

**Table 1:** Clinical data from 92 acromegalic patients over a 28-year follow-up period

Variable	Value
Female n (%)	66 (71.7)
Age (years)	58 (22)
BMI (kg/m²)	30 (8)
Overweight n (%)	29 (34.1)
Obesity n (%)	44 (51.8)
Prediabetes n (%)	29 (31.5)
Diabetes n (%)	40 (43.4)
Hypertension n (%)	53 (57.6)
Macroadenoma n (%)	75 (81.5)
Only surgery n (%)	29 (31.5)
Only medication n (%)	7 (7.6)
Surgery plus medication n (%)	53 (57.6)
No surgery or medication n (%)	3 (3.3)
Follow-up (years)	13 (10)
Annual control percentage	50 (52.7)
Initial IGF-I/LSN	3.30 (2.0)
Final IGF-I/LSN	0.86 (0.6)

BMI: body mass index; IGF-I/ULN (insulin-like growth factor-I/upper limit of normal). Values are expressed as n (%), and as median (interquartile distance), for age, BMI, follow-up, control percentage, and initial and final IGF-I/ULN

The annual control percentage was defined as the average of the ratios between each of the 969 IGF-I measurements over the total follow-up period and the respective upper age limit (IGF-I/ULN) less than 1

Table 2 shows the activity of disease and the treatment at the last consultation. Acromegaly was rated as inactive in 67.4% of patients. Of the patients with inactive disease, 38 (41.3%) were not taking any acromegaly related medication at the final assessment, and three of them had received radiotherapy.

**Table 2:** Acromegaly activity in 92 patients and treatment at the last visit

Treatment	Active	Inactive
CB	0	3 (3.3)
PAL	2 (2.2)	1 (1)
OCT or LAN	8 (8.7)	20 (21.7)
CB + OCT	5 (5.4)	0
CB + LAN	4 (4.3)	0
CB + PAL	1 (1)	0
CB + LAN + PEG	1 (1)	0
No treatment	9 (9.8)	38 (41)
Total	30 (32.6)	62 (67.4)

Data are expressed as n (%). Disease activity was defined when the ratio between each patient’s last IGF-I measurement and their respective upper age limit (IGF-I/ULN) was greater than 1

CB: cabergoline; PAL: paltusotine; OCT: octreotide; LAN: lanreotide; PEG: Pegvisomant

Table 3 shows thyroid carcinoma frequency cases in 92 patients with 4 cases diagnosed (4.3%). A patient presented with both medullary thyroid carcinoma (MTC) and papillary thyroid carcinoma (PTC); in this case, the MTC was a histopathological finding, presenting as a microscopic tumor of 4 millimeters, with normal serum calcitonin. Only one case of cancer occurred in a patient with active disease.

**Table 3:** Thyroid cancer prevalence in 92 acromegalic patients

Patient	Age (years)	Sex	% control	Histopathology	ATA Risk
1	42	Fem	83.3	Papillary	Medium-high
2	44	Fem	75.0	Papillary	High
3	72	Fem	12.5	Papillary + Medullary	High
4	43	Fem	66.7	Papillary	Low

Medullary cancer was a histopathology finding in case 3

Table 4 shows sonographic findings and thyroid function in 74 patients., In some cases more than one finding was present in the same patient. Seventeen patients showed no ultrasound abnormalities.

**Table 4:** Thyroid changes in 74 patients who underwent ultrasound

Characteristics	n (%)
No sonographic changes	17(23.0)
Echotextural changes	18(24.3)
Thyroid nodules	47(63.5)
Goiter*	20(27)
Primary hypothyroidism	2(2.7)
Central hypothyroidism	22(29.7)
Hyperthyroidism	2(2.7)

Some characteristics overlapped in the same patient.

*Thyroid volume reference: female 10–15 cm³ and male 12–18 cm³ [55]

Table 5 shows the size of thyroid nodules (largest nodule dimension) according to acromegaly activity in 74 patients who underwent ultrasound. Most nodules were less than 1 cm and were found in patients with inactive disease (*p* = 0.038 by chi-square test).

**Table 5:** Thyroid nodule size (largest nodule dimension) according to acromegaly activity in 74 patients undergoing ultrasound

Size of nodule	Active n (percentage)	Inactive n (percentage)
No nodules	11 (52.4)	15 (28.3)
≤ 1 cm	5 (23.8)	30 (56.7)
> 1 cm	5 (23.8)	8 (15.0)
Total	21 (100)	53 (100)

The p value by Pearson’s chi-square test was = 0.038

Activity was defined as the ratio between the last IGF-I measurement and the respective upper age limit, greater than 1

Table 6 shows the Bethesda System for Thyroid Cytopathology Reporting in 10 thyroid nodules submitted to Ultrasound-Guided Fine Needle Aspiration Cytology (FNAC) according to ACR-TIRADS criteria, with a malignancy risk ≤ 3% in 60% of cases.

**Table 6:** Bethesda System score* in 10 thyroid nodules submitted to a FNAB

Bethesda*	Number of nodules ( percentage)
1	1 (10)
2	6 (60)
3	2 (20)
4	1 (10)
5	0 (0)
6	0 (0)
Total	10 (100)

*Bethesda System for Reporting Thyroid Cytopathology, third edition 2023 [56]

Table 7 shows the disease status according to treatment modality and presence of diffuse thyroid hyperplasia in 74 patients. There was no difference in the percentage of disease activity between the presence (36.8%) and absence of diffuse thyroid hyperplasia (25.5%).

**Table 7:** Disease status, n (%); according to treatment modality and presence of diffuse thyroid hyperplasia in 74 patients who underwent ultrasound, according to treatment

Treatment	Diffuse thyroid hyperplasia		No diffuse thyroid hyperplasia	
	Active 7 (36.8)	Inactive 12 (63.2)	Active 14 (25.5)	Inactive 41 (74.5)
SRLs	1 (14.3)	2 (16.7)	0	1 (2.4)
SRLs + CB	0 (0)	0(0)	1 (7.1)	0(0)
TSA + CB	0(0)	0(0)	0(0)	2 (4.9)
TSA/TCC + SRLs	1 (14.3)	3 (25)	4 (28.6)	12 (29.3)
TSA/TCC + CB + SRLs	0(0)	0(0)	4 (28.6)	2 (4.9)
TSA + PEG + CB + SRLs	1 (14.3)	0(0)	0(0)	0(0)
TSA/TCC and/or RT	3 (42.9)	7 (58.3)	2 (14.3)	21 (51.2)
TSA + RT + CB	0(0)	0(0)	0(0)	1 (2.4)
TSA + RT +SRLs	0(0)	0(0)	1 (7.1)	0(0)
TSA/TCC + RT +SRLs + CB	0(0)	0(0)	1 (7.1)	1 (2.4)
No treatment or apoplexy	1 (14.3)	0(0)	1 (7.1)	1 (2.4)

Diffuse thyroid hyperplasia: volume > 15 cm³ in women and > 18 cm³ in men [reference 51]. TSA: transsphenoidal adenomectomy; TCC: transcranial surgery; RT: radiotherapy; CB: cabergoline; SRLs: somatostatin receptor ligands

PEG: pegvisomant

Positive correlations were found between control percentage and following time (*r* = 0.378; *p* = 0.001), and between control percentage and age (*r* = 0.318; *p* = 0.006). On the other hand, there was a negative correlation trend between thyroid volume and control percentage (*r* = -0.207; *p* = 0.077).

Table 8 shows the cluster analysis of the 74 patients who underwent ultrasound. Three distinct groups were segregated with highly significant differences between them in relation to sex and echotextural changes (*p* < 0.0001 in both cases). Age, number of nodules, thyroid volume and follow-up time are similar between the clusters. although the percentage of control showed a decreasing trend from group 1 to other groups.

**Table 8:** Cluster data in 74 acromegalic patients with in situ thyroid, with 18 patients in cluster 1, 36 patients in cluster 2, and 20 patients in cluster 3

Variables	Cluster 1	Cluster 2	Cluster 3	*p*
Sex Female (%)	(83.3)	(100)	0 (0)	< 0.0001
Echotextural changes (%)	100	0	0	< 0.0001
Age (years)	64 (22.2)	60 (20)	52.5 (23.2)	0.25
Nodules (n)	2 (3.5)	2 (3)	0 (2.7)	0.35
Thyroid volume (cm³)	10.3 (8.3)	10.3 (6.2)	12.9 (12.5)	0.57
Follow up time (years)	12.5 (11.2)	14 (11)	14 (12)	0.59
Control percentage	72.5 (36.8)	50 (67.3)	33.3 (62.6)	0.07

Value expressed in median (IQR), except for ecotextural alteration, expressed in n (%)

## Discussion

In the first part of this study, we have found that 70% were women and approximately 81.5% of cases were due to macroadenomas. Three-quarters of them were pre-diabetic or diabetic, and three-fifths had hypertension. These data are, for the most part, similar to most published clinical-epidemiological data, with the exception of sex, which generally shows an almost equal distribution in acromegaly [11, 12, 14, 34–37]. In two articles from Brazil, the women’s predominance was 58.5% (32) and 61.3% (33), lower than in the present study. It is known that women tend to have a greater delay in diagnosis compared to men (14). Although it is possible that modern increased awareness of the disease may have reduced this delay thereby increasing the frequency of acromegaly diagnosis in women, the explanations for the high frequency of women in our study are not yet clear.

Across the entire follow-up period, the average rate of control was 50%, and the percental of inactive disease at the lats examination was 70%, using the criterion of IGF-I within normal range in accordance with the concept that maintaining serum IGF-I levels in the middle to upper half of the age-related reference range is recommended [38, 39]. In this study, we chose not to address the debate on biochemical control, remission, and cure of acromegaly, nor did we analyze temporal trends. Instead, we used a practical and robust metric that indicates control during follow-up and disease activity at the last visit. Of the 92 patients, 4.3% had thyroid cancer, primarily papillary carcinoma. The pooled prevalence of thyroid cancer in acromegaly was about 3% in studies published before 2008, and about 6% in studies published since then [35, 40]. As previously stated, IGF-I is important additional factor, collaborating with TSH in regulating thyroid proliferation and differentiation [5, 6, 7, 9, 41]. In human thyroid carcinomas from non-acromegaly patients increased expression of both IGF-I and the IGF-I receptor has been demonstrated, associated with the upregulation of IGF-I mRNA. The immunoreactivity of both correlates positively with tumor diameter and intrathyroidal extension, but not with patient sex and age, nor with tumor stage or the occurrence of lymph node metastases [41]. Although the progression from a single normal cell to a fully malignant phenotype requires both the activation of oncogenes and the inactivation of tumor suppressor genes, paracrine and/or autocrine growth pathways can regulate neoplastic transformation. In this sense, understanding the biological mechanisms involved in thyroid cancer progression is important for developing new effective prognostic or therapeutic approaches [41].

Our data seems to agree with the absence of recommendation of an intensive thyroid cancer screening in acromegaly. In other words, patients with acromegaly should undergo regular examinations with hormonal and ultrasound evaluation of the thyroid and fine-needle aspiration biopsy when necessary, similarly to the general population [40]. In fact, reports of thyroid cancer in patients with acromegaly may be exaggerated, due to the rigorous and prolonged monitoring of these patients [42].

In the second experiment, about 63% of the 74 individuals who underwent thyroid ultrasound presented with thyroid nodules and one-fifth presented diffuse thyroid hyperplasia. In previous studies, thyroid nodules were reported on ultrasound in 43% to 75.6% of patients with acromegaly, with a pooled prevalence of 59.2%, similar to our findings [26, 35]. Thyroid gland volume and frequency of nodules were associated in several, but not all, studies with disease duration and GH and IGF-I levels [43, 44, 45, 46]. Two Brazilian studies published more than a decade ago suggested a higher frequency of diffuse or nodular goiter and thyroid cancer than in the general population [30, 31]. Conversely, in the present study, there was no association between the presence of diffuse thyroid hyperplasia and disease activity. Furthermore, most nodules were less than 1 cm and were found in patients with inactive disease, suggesting a micronodular disease pattern that appears to have less clinical relevance and that is spreading widely around the world [47].

On the other hand, only one case of cancer occurred in a patient with active disease. This raises the question of the residual effect of excess GH on the thyroid gland after biochemical remission in patients with acromegaly, the “legacy effect”. It is generally accepted that diffuse hyperplasia, but not thyroid nodules, can be reversed in cured acromegaly [48]. Unfortunately, we do not have a sufficient number of thyroid cancer cases to speculate on whether a past exposure to excessive GH may contribute to the progression of a nodule to thyroid cancer in patients with controlled acromegaly.

Our patients come from 2 very different areas: the large, landlocked Brazilian state of Minas Gerais is the fourth largest in Brazil, covering an area of 586,000 km², and borders seven other states. It is the second most populous state in the country, with approximately 21 million inhabitants, and has a diverse ethnic composition, with 46.8% identifying as mixed-race, 41.1% as white, and 11.8% as black. The low-lying, coastal state of Sergipe is the smallest in Brazil, with an area of approximately 21,938 km² and a population of about 2.21 million inhabitants, the majority of whom identify as mixed-race (61.6%), white (25.2%), and black (12.8%).

Currently, iodine deficiency is not considered an endemic public health problem in either state, thanks to the success of the salt iodization program instituted in 1953. Therefore, neither ethnicity nor iodide intake should influence our results. On the other hand, although we did not specifically address smoking, the prevalence of smoking in the adult population decreased from 35.3% to 11.3% in Brazil between 1990 and 2017 [49]. This may have reduced the impact of smoking on the studied cases. Similarly, family history was reported only in two first-degree relatives of the patients.

As mentioned earlier, cluster analysis identifies groups in the data when explanatory variables are provided. It disaggregates a set of objects into clusters whose number is not previously specified and whose characteristics are randomly determined. This analysis seems to indicate that the distribution of patients into the clusters is not influenced by the state of acromegaly but rather is defined by the characteristics of the studied population itself, as it does not demonstrate an association between acromegaly and the number of thyroid nodules or thyroid volume.

First of all, there was no difference in the frequency of nodules and in the thyroid volume among the three clusters, despite the median percentage of control reduced successively from cluster 1 to cluster 3. Sex and echotextural changes were the best discriminators among the clusters. Although acromegaly is diagnosed more frequently in women, sex is not a modifiable risk factor for acromegaly. Notably, the frequency of these ecotextural alterations was diametrically opposed in two predominantly female groups, being universal in group 1, which had a higher rate of good control, and absent in group 2, exclusively female, with an apparently smaller percentage of control. Cluster 2 has an apparently lower control rate, but has the same thyroid volume as cluster 1.These data indicate that these characteristics, sex and echotextural changes have little or no relation to acromegaly, at least in these 74 Brazilian individuals. These ecotextural changes may be related to susceptibility determined by genetic factors (such as predisposition to Hashimoto thyroiditis) [50] and do not appear to be inherent to acromegaly. Accordingly, the frequency of hyperthyroidism in the present study (2.7%) was also lower than the previously reported between 5.0 and 14.7% [45, 51, 52].

We found a positive correlation between control percentage and following time and a negative correlation trend between thyroid volume and control percentage. This suggests that prolonging acromegaly treatment with current drugs may mitigate the consequences of somatotropic axis hyperfunction on the thyroid. The natural history of acromegaly seems to have changed with treatment evolution and more biochemical control resulting in apparently less thyroid findings. These advancements have also resulted in a mortality rate that is now comparable to that of the general population in controlled patients [11, 12].

One limitation of this study was the absence of a control group without acromegaly. In any case, the frequency of thyroid nodules in our study was similar to other Brazilian populations evaluated by ultrasound [47, 53, 54]. However, this comparison is also subject to biases related to the quality of the records and the epidemic of diagnosis due to the high use of thyroid ultrasonography. To minimize this aspect, we focused on the degree of control throughout the patients’ follow-up period. Another possible criticism of our data would be the lack of evaluation of iodine status; however, as mentioned above, iodine has been added to table salt in Brazil for several decades. On the other hand, a strength of this work is the fact that the ultrasound examination was performed in the transversal part of the study by a single examiner in all patients.

In conclusion, no inherent thyroid changes were apparently identified in a large cohort of Brazilian patients with acromegaly, followed for almost 30 years to date. Reducing the time to diagnosis and strict hormonal control during follow-up may attenuate thyroid manifestations in acromegaly.

## Data Availability

All current data are available from the lead investigator (MHA-O) and can be provided upon request.
